# Supplementary material for: Hierarchical and nonhierarchical features of the mouse visual cortical network
Source: Nat Commun. 2022 Jan 26;13:503. doi: 10.1038/s41467-022-28035-y (PMC8791996; doi:10.1038/s41467-022-28035-y)
Supplement: Supplementary file 1 — Supplementary Information [file 41467_2022_28035_MOESM1_ESM.pdf]

SUPPLEMENTARY FIGURES

Supp. Fig. 1

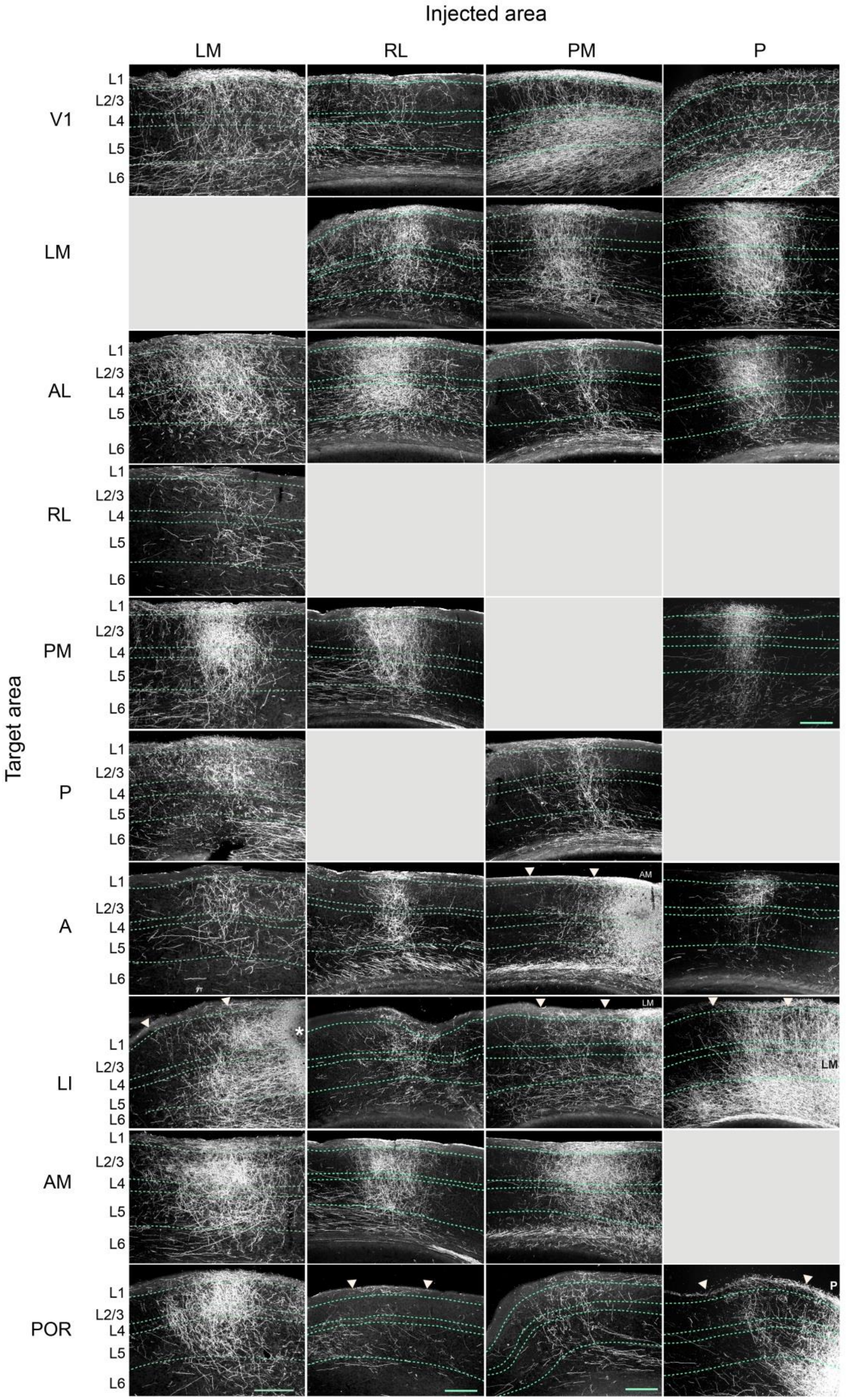

Supplementary Fig. 1: Interareal termination patterns of anterogradely labeled projections from areas LM, RL, PM, and P

Representative dark-field images of anterograde BDA labeled axonal projections from LM, RL, PM, and P to each of the other nine areas in which terminations were sufficiently dense to allow analysis. In L1-4 used for the analysis of projection density, axons preferentially target L1 of V1, but show varying preferences in the other areas. White asterisk in the LM→LI panel denotes region in LM near injection site. White arrowheads in the LM→LI, RL→POR, PM→A, PM→LI, P→LI, and P→POR panels demarcate the respective target area used for analysis. Scale bars, 200 µm.

Supp. Fig. 2

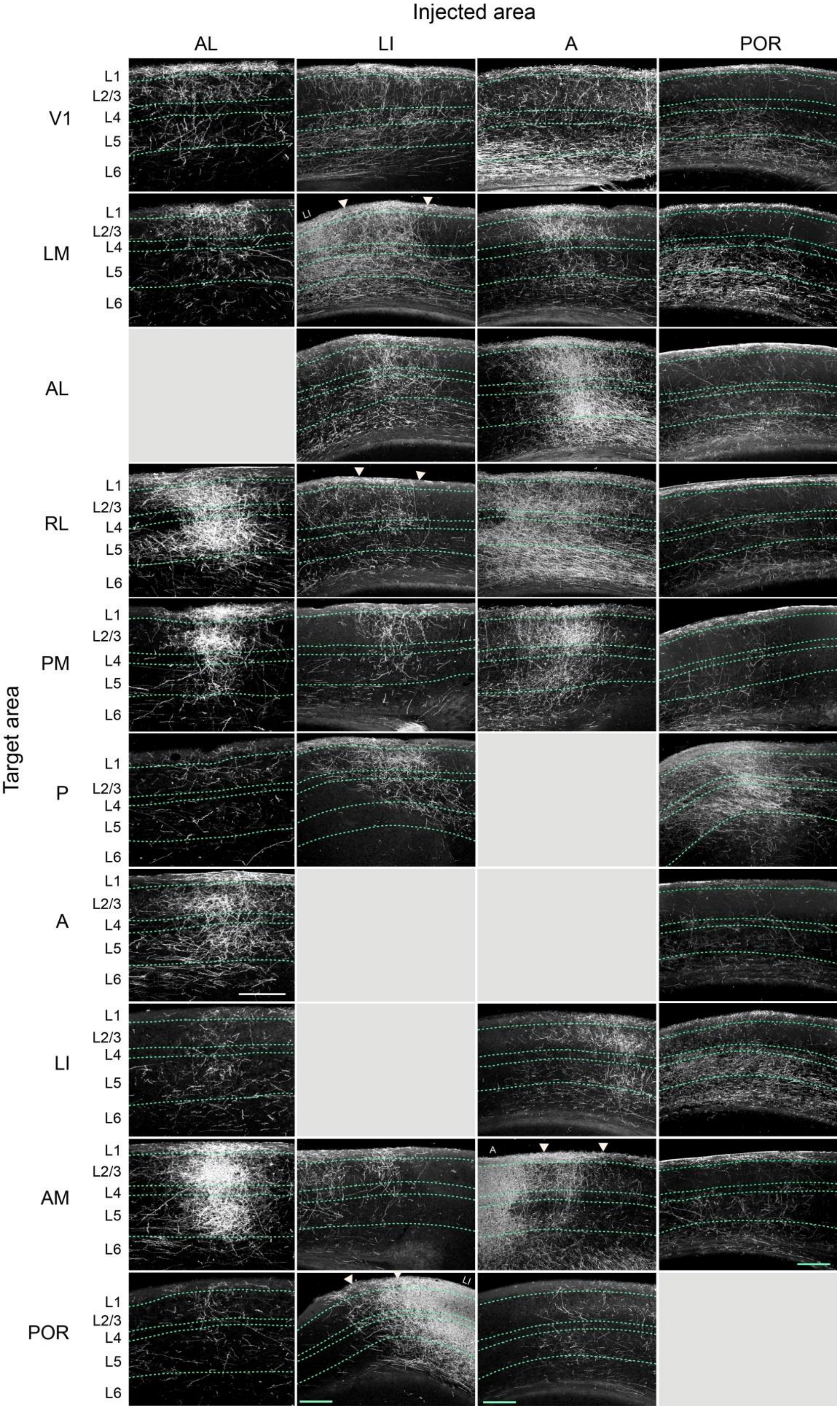

Injected area
AL
LI
A
POR
Target area
V1
LM
AL
RL
PM
P
A
LI
AM
POR
L1
L2/3
L4
L5
L6

Supplementary Fig. 2: Interareal termination patterns of anterogradely labeled projections from areas AL, LI, A, and POR

Representative dark-field images of anterograde BDA labeled axonal projections from AL, LI, A, and POR to each of the other nine areas in which terminations were sufficiently dense to allow analysis. White arrowheads in the LI→LM, LI→RL, LI→POR, and A→AM panels demarcate the respective target area used for analysis. Scale bars, 200 μm.

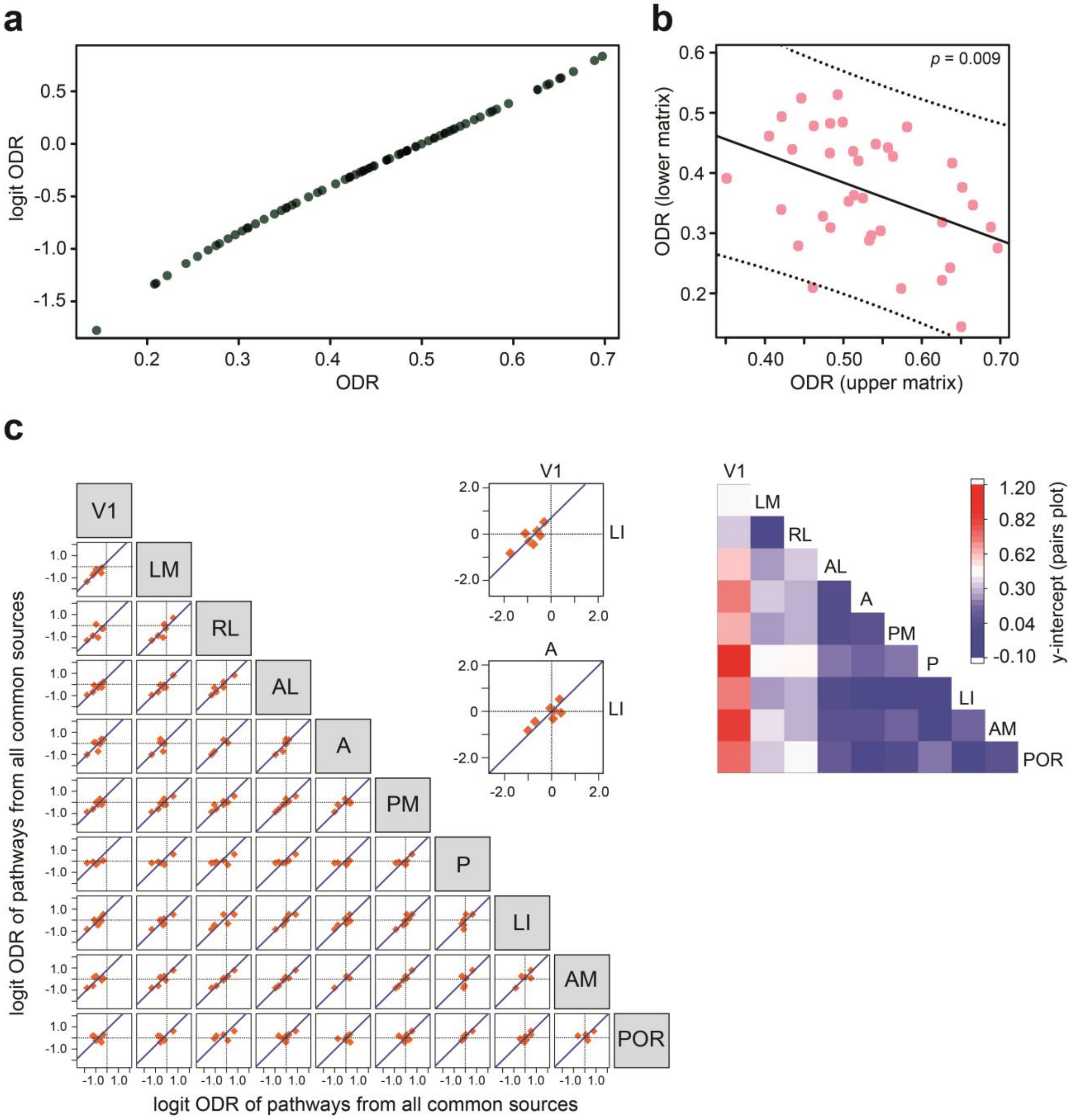

Supplementary Fig. 3: Scatterplots of logit ODRs of pathways from all common source areas to pairs of target areas

a. The logit of each ODR plotted against the corresponding ODR value.

b. The ODR for each pathway plotted against that of its reciprocal counterpart for all 74 pathways that have a dense reciprocal connection (see Fig. 3a). 'Upper matrix' and 'lower matrix' refer respectively to the ODRs in the upper/right and lower/left triangular halves of the matrix in Fig. 3a. The fit shows a significant negative association (slope = -0.48, p = 0.009, F-test).

c. Scatterplots showing the correlation of logit ODR values for pathways originating in all common sources (injected areas) for any two target areas. The horizontal axis of each plot corresponds to the logit ODR of the pathway terminating in the area indicated at the top of each column, and the vertical axis corresponds to the logit ODR of the pathway terminating in the area indicated at the right of each row. Thus, each orange point plots against each other the logit ODRs for pathways that originate in a common source area that terminates in the two indicated target areas. Dotted lines, coordinate axes. A line of unit slope (blue) that best fit the orange points is plotted in each graph, and the absolute value of the y-intercept of this unit line provides a measure for hierarchical distance between the two indicated target areas. Two example graphs are shown at higher magnification with the target areas indicated. Note that the absolute value of the y-intercept in the graph plotting pathways terminating in V1 and LI (y-intercept, 0.741) is greater than that in the graph for pathways terminating A and LI (y-intercept, -0.006). This indicates that V1 and LI are more hierarchically distant than are A and LI when only projections terminating in these three areas are considered. The y-intercepts of each graph are plotted as a heat map on the right.

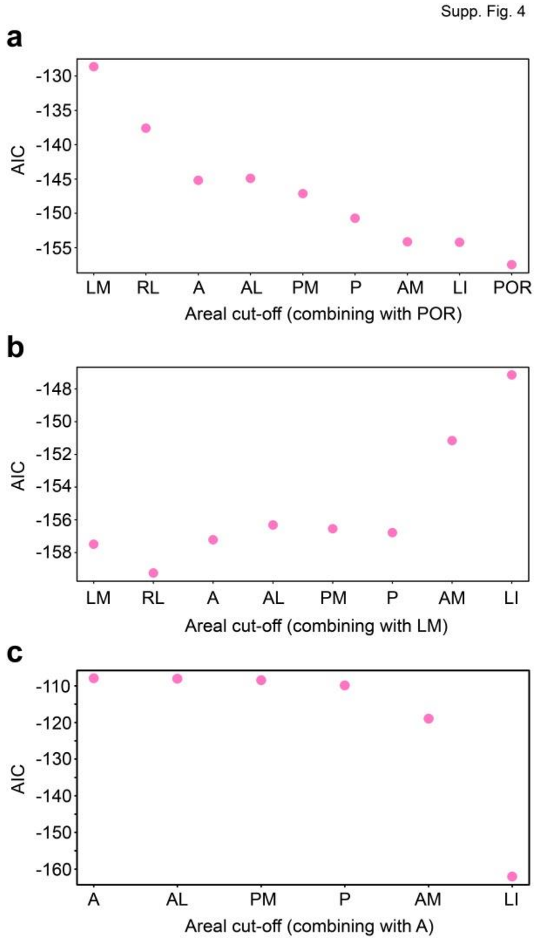

Supplementary Fig. 4: AIC scores of various hierarchical models

a. The AIC values for nine models in which the highest n areas (n going from 1 to 9, beginning with the highest area POR) were constrained to be part of the same level, and the beta regression fit performed for each such model. The lowest AIC value occurs for the model in which all areas from LM to POR are at separate levels, indicating that a model with POR at a separate level is better than any model that combines lower levels with it.

b. The AIC values for eight models in which the lowest n areas (n going from 1 to 8, beginning with the lowest area LM) were constrained to be part of the same level, and the beta regression fit performed for each such model. The lowest AIC value occurs for the model in which areas LM and RL are considered to be at the same level, with all other areas at different levels. This model also has a lower AIC value than the one with lowest value in Supplementary Fig. 4a.

c. The AIC values for six different models in which LM and RL are considered to be at the same hierarchical level, with areas higher than the LM/RL level and those lower than POR systematically fused into one level. The six models were generated by successively and cumulatively merging areas to A. The lowest AIC score occurs for the model in which areas A to LI were combined into one level, resulting in a hierarchy of four levels (V1, LM/RL, A/AL/PM/P/AM/LI, and POR) as the best model of those tested here.

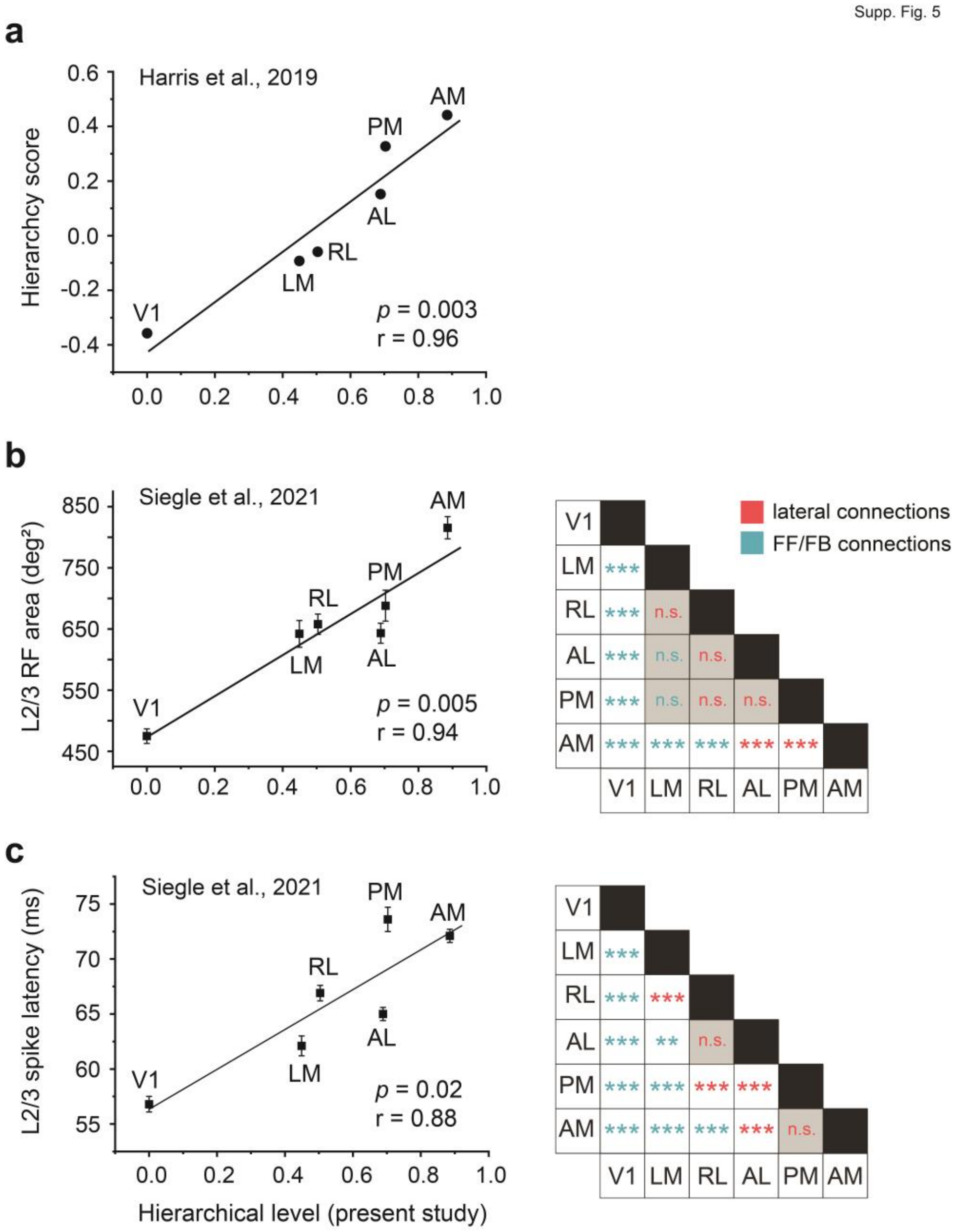

Supplementary Fig. 5: Correlation of hierarchical organization with open-source physiological data obtained from awake, head-fixed mice

a. The hierarchical levels of the present study are significantly correlated with the 'hierarchy score' determined in Harris et al., 2019[14].

b. *Left*. RF sizes of L2/3 neurons recorded in awake, head-fixed mice are significantly correlated with hierarchical level (p = 0.005, Pearson correlation coefficient (r) = 0.94).

Right. Pairwise comparisons of RF areas indicate that 11/15 pairs show a consistent relationship between differences in RF sizes and the hierarchical nature (i.e., whether FF/FB or lateral) of the

pathways connecting the corresponding areal pair (n.s., not significant; ***p < 0.001; two-sided t-test with Holm-Bonferroni correction).

c. *Left*. The time to first spike in L2/3 neurons recorded in awake, head-fixed mice are significantly correlated with hierarchical level ($p = 0.02$, Pearson correlation coefficient (r) = 0.88).

Right. Pairwise comparisons of the time to first spike indicate that 11/15 pairs show a consistent relationship between differences in the time to first spike and the hierarchical nature of the pathways connecting the two areas (n.s., not significant; **p < 0.01; ***p < 0.001; two-sided t-test with Holm-Bonferroni correction).

Data in b and c obtained from Siegle et al., 2021[6].

Supp. Fig. 6

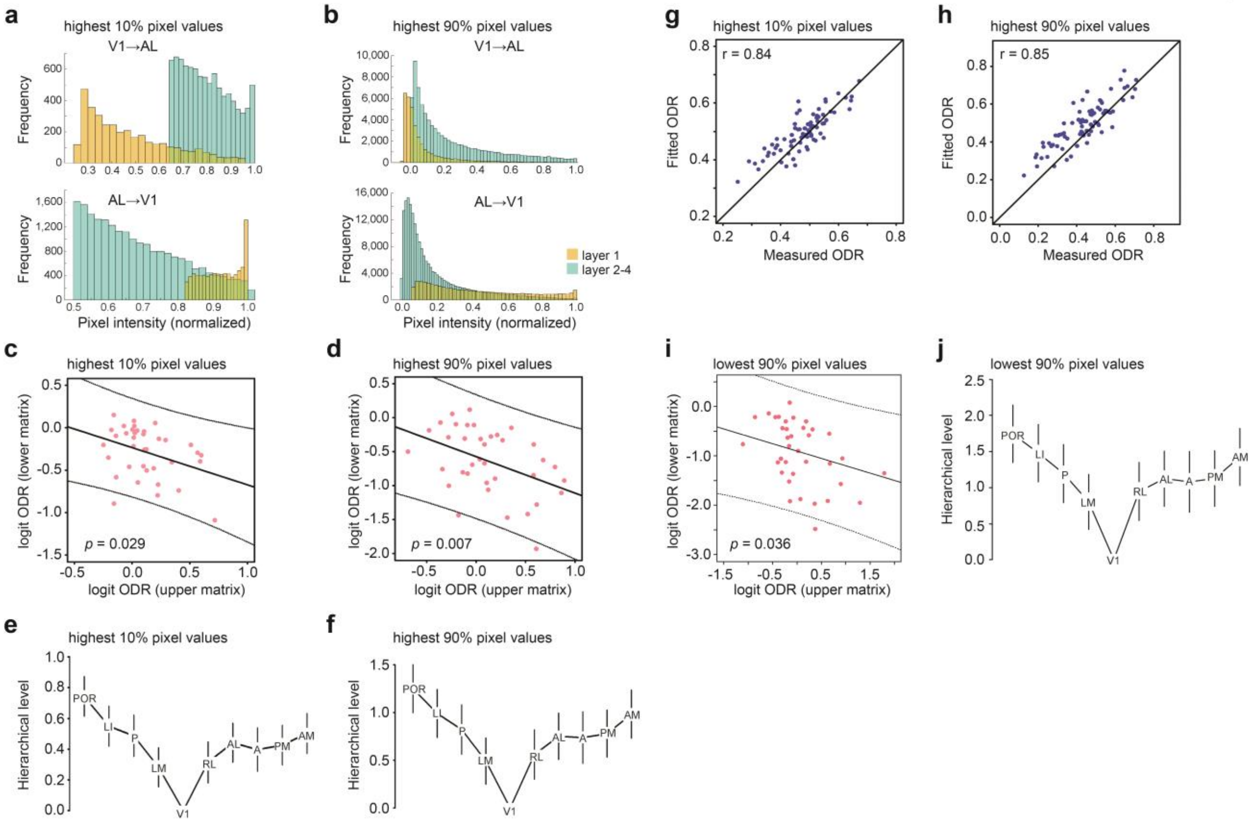

Supplementary Fig. 6: Hierarchical properties show modest changes across selection of pixel intensity values

a. Distribution of pixel intensity values in L1 (yellow) and L2-4 (blue) of representative termination patterns of V1→AL and AL→V1 pathways (same grayscale images as in Fig. 2a). Only pixels within the highest 10% intensities were included. Average background intensity has been subtracted from all pixel values.

b. Similar histogram plot as in Supplementary Fig. 6a, but only with pixels within the highest 90% intensities included. Average background intensity has been subtracted from all pixel values.

c. Logit ODR for each pathway plotted against that of its reciprocal counterpart for all 74 pathways that have a dense reciprocal connection, after only pixels with the highest 10% intensities were included in the analysis. Upper matrix and lower matrix refer respectively to the ODR values in the upper/right and lower/left triangular halves of a matrix similar to that shown in Fig. 3a, but for ODRs after selection of only the brightest 10% pixels. The fit shows a significant negative association (slope = -0.44, p = 0.029, F-test) indicating that the more FF a pathway is in one direction, the more FB is the reciprocal pathway.

d. Similar plot as Supplementary Fig. 6c but with pixels with intensities within the highest 90% values included in the analysis (slope = -0.54, p = 0.007, F-test).

e. Estimated hierarchical levels obtained by a beta regression model after including only pixel values within the highest 10% intensities. Hierarchical level value of V1 is set at 0, and differences between any two hierarchical level values best predict the ODR for the pathways connecting the respective areas. Vertical lines demarcate 90% confidence intervals.

f. Similar hierarchical diagram as Supplementary Fig. 6e, but after inclusion of all pixels within the highest 90% intensities.

g. ODRs estimated by the beta regression model plotted against the measured ODRs show a high goodness of fit ($r = 0.84$) when only the highest 10% pixel values were included.

h. ODRs estimated by the beta regression model plotted against the measured ODRs show a high goodness of fit ($r = 0.85$) when only the highest 90% pixel values were included.

i. Logit ODR for each pathway plotted against that of its reciprocal counterpart for all 74 pathways that have a dense reciprocal connection, after only pixels with the lowest 90% intensities were included in the analysis. The fit shows a significant negative association (slope = -0.30, $p = 0.036$, F-test) indicating that greater the FF nature of a pathway is in one direction, greater is the FB nature of the reciprocal pathway even when pixels with the highest 10% values are eliminated.

j. Estimated hierarchical levels obtained by a beta regression model after including only pixel values within the lowest 90% intensities. Hierarchical level value of V1 is set at 0, and differences between any two hierarchical level values best predict the ODR for the pathways connecting the respective areas. Vertical lines demarcate 90% confidence intervals.
