# Supplementary material for: Hierarchical and nonhierarchical features of the mouse visual cortical network
Source: Nat Commun. 2022 Jan 26;13:503. doi: 10.1038/s41467-022-28035-y (PMC8791996; doi:10.1038/s41467-022-28035-y)
Supplement: Supplementary file 3 — Reporting Summary [file 41467_2022_28035_MOESM3_ESM.pdf]

nature portfolio

Corresponding author(s): Andreas Burkhalter

Last updated by author(s): Dec 2, 2021

Reporting Summary

Nature Portfolio wishes to improve the reproducibility of the work that we publish. This form provides structure for consistency and transparency in reporting. For further information on Nature Portfolio policies, see our Editorial Policies and the Editorial Policy Checklist.

Statistics

For all statistical analyses, confirm that the following items are present in the figure legend, table legend, main text, or Methods section.

n/a	Confirmed	
☐	☒	The exact sample size (*n*) for each experimental group/condition, given as a discrete number and unit of measurement
☐	☒	A statement on whether measurements were taken from distinct samples or whether the same sample was measured repeatedly
☐	☒	The statistical test(s) used AND whether they are one- or two-sided<br>*Only common tests should be described solely by name; describe more complex techniques in the Methods section.*
☒	☐	A description of all covariates tested
☐	☒	A description of any assumptions or corrections, such as tests of normality and adjustment for multiple comparisons
☐	☒	A full description of the statistical parameters including central tendency (e.g. means) or other basic estimates (e.g. regression coefficient) AND variation (e.g. standard deviation) or associated estimates of uncertainty (e.g. confidence intervals)
☐	☒	For null hypothesis testing, the test statistic (e.g. *F*, *t*, *r*) with confidence intervals, effect sizes, degrees of freedom and *P* value noted<br>*Give P values as exact values whenever suitable.*
☒	☐	For Bayesian analysis, information on the choice of priors and Markov chain Monte Carlo settings
☐	☒	For hierarchical and complex designs, identification of the appropriate level for tests and full reporting of outcomes
☐	☒	Estimates of effect sizes (e.g. Cohen's *d*, Pearson's *r*), indicating how they were calculated

Our web collection on statistics for biologists contains articles on many of the points above.

Software and code

Policy information about availability of computer code

Data collection	The TEMPO software (Tempo, Reflective Computing) was used to collect electrophysiology data. MetaMorph (ver. 7.8.12.0), ImageJ (v1.53c), and MATLAB (R2020a) were used to generate images for analysis.
Data analysis	Statistical tests for generating and analyzing the cortical hierarchy were performed using the Open Source software R with the betareg package (ver. 3.1-4). Custom-written code used to analyze data is available at github.com/rdsouza2882/NatComm2021

For manuscripts utilizing custom algorithms or software that are central to the research but not yet described in published literature, software must be made available to editors and reviewers. We strongly encourage code deposition in a community repository (e.g. GitHub). See the Nature Portfolio guidelines for submitting code & software for further information.

Data

Policy information about availability of data

All manuscripts must include a data availability statement. This statement should provide the following information, where applicable:

- Accession codes, unique identifiers, or web links for publicly available datasets
- A description of any restrictions on data availability
- For clinical datasets or third party data, please ensure that the statement adheres to our policy

Logit ODR data for all pathways are available at github.com/rdsouza2882/NatComm2021. Additional data that support the findings of this study are available from the corresponding author (A.B.) upon reasonable request.

Field-specific reporting

Please select the one below that is the best fit for your research. If you are not sure, read the appropriate sections before making your selection.

☒ Life sciences ☐ Behavioural & social sciences ☐ Ecological, evolutionary & environmental sciences

For a reference copy of the document with all sections, see nature.com/documents/nr-reporting-summary-flat.pdf

Life sciences study design

All studies must disclose on these points even when the disclosure is negative.

Sample size	Sample sizes for anatomy and electrophysiology experiments were based on previous experience and other studies in the field (Niell and Stryker, Neuron 2010, doi: 10.1016/j.neuron.2010.01.033; Wang et al., J Neurosci 2011, doi: 10.1523/JNEUROSCI.3488-10.2011; Wang et al., J Neurosci 2012, doi: 10.1523/JNEUROSCI.6063-11.2012; Gamanut et al., Neuron 2018, doi: 10.1016/j.neuron.2017.12.037; Horvat et al., PLoS Biol 2016, doi: 10.1371/journal.pbio.1002512).
Data exclusions	No data were excluded from analysis. Brain sections in which terminal axonal projections contained retrogradely labeled cells were excluded from analysis.
Replication	Optical density ratios were obtained after averaging 3 to 5 sections for each anterogradely labeled pathway, with two injections per pathway. All attempts at replication were successful. Injections where the site of injection was ambiguous or if the injection labeled multiple areas were excluded from analysis. No other injections were performed. Analyses were repeated with different thresholds of optical densities for the inclusion of labeled axons.
Randomization	Areas were randomly selected for injection and recordings; injections and recordings were performed in each of the ten areas in no particular order. For injections, male and female animals were allocated such that each area was injected two times (one injection per animal). All animals used in the study were adults with all experiments performed after the critical period. Controlling for age was therefore not considered relevant to the study.
Blinding	Blinding was not performed for injections or recordings because areas were randomly selected a priori, and injections performed using stereotaxic coordinates, with areal locations/identities confirmed post hoc using anatomical landmarks. The hierarchy analysis was performed on the 10x10 matrix of density ratios, blinded to the density ratio of each pathway.

Reporting for specific materials, systems and methods

We require information from authors about some types of materials, experimental systems and methods used in many studies. Here, indicate whether each material, system or method listed is relevant to your study. If you are not sure if a list item applies to your research, read the appropriate section before selecting a response.

Materials & experimental systems

n/a	Involved in the study	
☐	☒	Antibodies
☒	☐	Eukaryotic cell lines
☒	☐	Palaeontology and archaeology
☐	☒	Animals and other organisms
☒	☐	Human research participants
☒	☐	Clinical data
☒	☐	Dual use research of concern

Methods

n/a	Involved in the study	
☒	☐	ChIP-seq
☒	☐	Flow cytometry
☒	☐	MRI-based neuroimaging

Antibodies

Antibodies used	bisBenzimide H 33258 (Sigma-Aldrich, catalog # B2883, CAS # 23491-45-4)<br><br>Anti-muscarinic acetylcholine receptor m2 antibody (Fisher Scientific, catalog # MAB367MI)<br><br>Anti-muscarinic acetylcholine receptor m2 antibody (EMD Millipore, catalog # MAB367, clone M2-2-B3)<br><br>Biotynilated dextran amine (BDA-10,000) (ThermoFisher, catalog # D1956)<br><br>Goat anti rat IgG Alexa Fluor 647 (ThermoFisher, catalog # A21247)
Validation	Each of the antibodies used in this study has been successfully used and validated in previous studies:<br><br>bisBenzimide H 33258: doi: 10.1523/JNEUROSCI.6063-11.2012

	Anti-muscarinic acetylcholine receptor m2 antibody: doi: 10.1016/j.neuron.2017.12.037; doi: 10.1016/j.neuron.2015.07.004<br>Biotynilated dextran amine (BDA-10,000): doi: 10.1523/JNEUROSCI.6063-11.2012; doi: 10.1523/JNEUROSCI.3067-12.2013<br>Goat anti rat IgG Alexa Fluor 647: doi: 10.1016/j.neuron.2015.07.004; doi: 10.1523/JNEUROSCI.3488-10.2011

Animals and other organisms

Policy information about studies involving animals; ARRIVE guidelines recommended for reporting animal research

Laboratory animals	5-16 weeks old C57BL/6J male and female mice
Wild animals	No wild animals were used in this study
Field-collected samples	No field collected samples were used in this study
Ethics oversight	All experimental procedures were approved by the Institutional Animal Care and Use Committee at Washington University in St. Louis. Mice were housed in cages (food, water, bedding, micro isolator bonnet) stacked on racks in a closed, climate controlled room (light cycle 12 h on/ 12 h off; ambient temperature 20-16 deg C; humidity 30-70%).

Note that full information on the approval of the study protocol must also be provided in the manuscript.
